# Managing adversity: a cross-sectional exploration of resilience in social care

**DOI:** 10.1186/s12877-025-06306-9

**Published:** 2025-09-03

**Authors:** Anita Mallon, Gary Mitchell, Gillian Carter, Derek F. McLaughlin, Corrina Grimes, Christine Brown Wilson

**Affiliations:** 1 School of Nursing and Midwifery, Queens University Belfast, University Rd, Belfast, Northern Ireland BT7 1NN Northern Ireland, UK; 2 Atlantic Fellow for Equity in Brain Health, Visiting Research Fellow Global Brain Health Institute, University of California San Francisco | Trinity College Dublin, Dublin, Ireland

**Keywords:** Domiciliary care, Residential care, Resilience, Social care, Socioecological theory, Work-related sense of coherence

## Abstract

**Background:**

The social care workforce in the United Kingdom plays a vital role in providing support services yet faces systemic challenges of lower priority and lack of resources. Resilience is crucial for these workers who have been navigating challenges within a fragmented system long before the onset of the COVID-19 pandemic. Understanding resilience factors is essential to the future proofing of this vital workforce.

**Methods:**

A quantitative, descriptive study was conducted using a cross-sectional online survey to assess resilience and associated factors of social care workers in Northern Ireland. The survey included the Connor-Davidson Resilience Scale (CD-RISC) and the Work-related Sense of Coherence Scale (Work-SoC), along with demographic and work-related questions. Data was collected from 613 participants through an online survey between May and June 2023.

**Results:**

Of the 613 valid responses analysed, the mean CD-RISC score was 70.4 (SD 13.3). Gender, education level, years of experience, age and Work-SoC showed varying impacts on resilience scores. For instance, females had higher resilience scores compared to males, and those with a bachelor’s degree and higher reported slightly increased resilience scores. Additionally, age categories influenced resilience scores, with older age groups exhibiting higher resilience levels. Findings revealed a moderate positive association between resilience and how people perceive their work in terms of its comprehensibility, manageability, and meaningfulness.

**Conclusion:**

The study highlights the importance of resilience among social care workers in Northern Ireland and emphasises the need to explore factors such as Work-related Sense of Coherence to enhance well-being and job performance. An all systems approach to building and sustaining resilience is proposed, recognising individual assets, fostering supportive work environments, and reshaping societal perception of social care.

**Supplementary Information:**

The online version contains supplementary material available at 10.1186/s12877-025-06306-9.

## Background

The social care workforce in the United Kingdom (UK) makes up a considerable proportion of employment [1]. Social care workers provide a wide and diverse range of support services to people in their own homes, residential care services and in community settings and as such are integral to a functioning health and social care service. As demand for these services increase, the job of social care workers has become more complex [2]. Reports from studies investigating the effects of the pandemic on those working outside the hospital environment in care homes and domiciliary care highlight a sense of lower priority, not only in terms of available resources but rather a systemic sense of having less value and worth than those based in hospital environments [3, 4]. Authors of two recent studies in Northern Ireland (NI) determining the impact of the pandemic on health and social care, highlight the need for future research to develop robust and strong pathways to interventions that will develop resilience and support wellbeing [5, 6]. Additionally, reviews of interventions aiming to support resilience of health care workers, advocate that attention should extend beyond the hospital to workers in social care [7, 8]. Despite the obstacles and prevailing crises in social care [9] recent qualitative research [10] has uncovered accounts of enduring compassion, exceeding expectations, and championing for individuals facing challenging situations among those employed in long-term care and community residential units.

Resilience may explain some of these assets and provide a pathway to the development of interventions that harness and capitalise on these attributes. Resilience in its true sense is a concept that refers to the presence of adversity and the ability for someone to not only recover but learn from and grow through the experience [11]. Unlike the concept of hardiness, that implies the ability to take on additional challenges and persevere, resilience is positive and adaptive [12]. The dynamic nature of resilience has gained increasing traction as something that includes adaptability, will change over the life span and is bidirectional from the individual and their environment [11, 12]. There is evidence to suggest the benefit of resilience to the individual, the organisation and society with reviews of empirical data suggesting that resilience can act as a buffer against burnout, secondary traumatic stress [13, 14] exhaustion, anxiety, and depression [15].

A pilot survey undertaken by the authors of this study investigating resilience in nurses employed in residential care/nursing homes [16] found that nurses working in residential facilities outside of hospital care in Northern Ireland reported a moderate level of resilience. Indeed, this correlates with a qualitative study of domiciliary care workers both before and after the pandemic who perceived themselves as highly resilient and adaptable [17]. Those who saw meaning in their work and felt they could make a difference were better able to manage and adapt in the face of added stressors [17]. In our survey, care home nurses’ demographics explained only a small amount of variance on resilience scores indicating that other factors undiscovered by these variables need to be explored [16]. While studies on resilience in hospital-based workers have proliferated over the last 10 years [18–21] there remains a dearth of research on supporting resilience of workers in care homes, domiciliary care, and other social care settings [4]. Understanding resilience and what factors underlie the resilient care worker, may go some way in the development of a pathway to interventions that will enable recovery and preparedness for future adversity.

Resilience as a concept is subject to diverse interpretations and in recent years has developed some negative connotations in its association with personal blame/weakness. Despite the debate there remains a lack of clarity of ‘underlying mechanisms’ which influence the development of resilience [22]. One mechanism that warrants further enquiry is an individual’s orientation to life or Sense of Coherence (SOC). This was first introduced by Antonosky [23], as a part of the salutogenic model of health. Salutogenesis may be understood as an umbrella term incorporating many concepts relating to assets for health and well-being such as resilience, self-efficacy, locus of control and sense of coherence [24]. It can be seen as a comprehensive socioecological systems theory of health [25], and therefore aligns with the socio-ecological perspective that underpins this study. The model proposes that experiences throughout life shape one’s orientation to life, one’s sense of coherence. People with a strong sense of coherence look for meaning in life and expect to find meaning in the challenges that they endure [23]. A strong sense of coherence facilitates the adaptive use of resources to tackle stressors [26]. It has been strongly associated with physical and mental health [27], and was found to be a protective factor against burnout in care home workers in Spain (n = 340) [28]. It differs from resilience in that adversity is not an essential characteristic but rather it refers to all of life experiences [26]. As an ecological concept any inquiry relating to resilience should also “explore the context in which the individual experiences adversity, making resilience first a quality of the broader social and physical ecology, and second a quality of the individual” (p. 27) [29] Antonosky also maintained the work environment is important in shaping one’s SOC [23].

As with any systems perspective each small part of the system needs to fit together to ensure the function of the whole system [30]. At an organisational level and a broader societal level, resilience and the well-being assets of the workforce are critical issues in maximising productivity, workability, retention, and job performance [31, 32]. The systems approach underpinning this study regards the work environment as crucial in shaping how an individual manages challenges, and how that may potentially impact their health. In a survey of psychological well-being among health and social care staff, the perception of less effective communication with the organisation predicted greater levels of anxiety, depression and post-traumatic stress and insomnia [5]. The embedded perspective of the socio-ecological model [33] would suggest then, that the health of the individual will impact other levels in the system such as the working environment and therefore requires both protection and nurturing.

Work-related Sense of Coherence (Work-SoC) maintains the ethos of the global Sense of Coherence scale [23] measuring the perceived comprehensibility (cognitive component identifies work as structured, consistent and clear) [34], manageability (instrumental component perception of resources that are available to cope with the demands of the job) [35], and meaningfulness as to whether an individual sees their work as worthy of commitment and involvement [35]. While research on this scale is in its infancy, a study exploring the incremental validity of the Work-SoC in predicting work wellness, found the Work-SoC to be a better predictor of both work engagement and fatigue than the SOC [36]. In a study determining the construct validity of the Work-SoC using both cross-sectional (*n* = 3412) and longitudinal (*n* = 1286) data, it was observed that having more job resources was linked to higher levels of Work-SoC while facing greater job demands was linked to lower levels of Work-SoC [34]. A recent longitudinal observational study of Swiss health care professionals (*n* = 1578) [37], found that those with higher levels of Work-SoC had less COVID anxiety, trauma, depression, and perceived vulnerability in the initial stages of the pandemic. Furthermore, Vogt et al. proposed that Work-SoC is a context specific application of the Sense of Coherence Scale, with levels remaining consistent regardless of demographic differences. The data suggests that work sense of coherence plays a significant role in the balancing of work-related stressors [34].

This survey addresses the recommendation from Ravalier’s study [6] that further enquiry is needed into the role of *positive* coping strategies in enhancing the well-being and work-related quality of life of the social care workforce. The study aims to make explicit the levels of resilience of social care workers in Northern Ireland thus providing a robust pathway and anchor for an all systems approach to understanding the resilience and associated factors of individuals working in social care.

This exploratory study answers the following research questions:

1. What are the resilience levels of social care workers in Northern Ireland?
2. To what extent do demographic factors and workplace characteristics shape resilience among social care workers?
3. What is the relationship between work sense of coherence and resilience among social care workers?

## Methods

### Study design

This study followed a quantitative, descriptive approach using a cross-sectional online survey.

### Study sample

The diversity and geographical spread of social care workers in NI required a multipronged recruitment process. Following a series of meetings with key stakeholders who agreed to disseminate and promote the study, a recruitment plan was devised to ensure that all registered social care workers, especially those working as care assistants and domiciliary care workers, would get the opportunity to participate. Registration with the Northern Ireland Social Care Council (NISCC) is a legal requirement for those working in social care roles across all service areas and sectors in Northern Ireland. There were 37,000 (approx.) registered social care workers in Northern Ireland at the time of the survey based on information provided by NISCC. A census sampling strategy was used whereby all social care workers who were registered with NISCC had the opportunity to participate. A power calculation was undertaken. Based on a margin error of 5% and a confidence level of 95% the minimum required sample to power the study for statistical significance was 381. Data collection took place between May and June 2023. The NISCC social media sites and the university social media were also used to boost recruitment. A reminder email was sent after two weeks, and a further social media post at four weeks.

### Procedure

The online survey was disseminated via an advertisement through the Northern Ireland Social Care Council (NISCC) newsletter emailed to all registrants. When respondents clicked on the link, they were directed to the survey site. The invite hosted the Participant Information Sheet (PIS) and screening questions to ensure eligibility to participate. To continue to the survey respondents had to confirm they had read the information sheet and wished to continue to the survey.

### Measures and materials

The survey contained 39 items and was designed using Qualtrics software. https://www.qualtrics.com/, It comprised three sections. Section 1; The Connor-Davidson Resilience Scale (CD-RISC) [38] Sect. 2; The Work-related Sense of Coherence Scale (Work-SoC) [34] and Sect. 3; Demographics and Work Characteristics. The guidelines for reporting cross-sectional studies (STROBE) were used to inform the reporting of this study [39]. A completed STROBE checklist is available in Supplementary File 1.

#### The Connor-Davidson scale (CD-RSIC) [38]

This is a 25-item scale used to measure resilience levels of participants. The scale was developed in general and clinical populations samples demonstrating internal consistency as demonstrated by a Cronbach’s alpha of. 89 in the general population and good test re-test reliability in the clinical population [38]. The CD-RSIC has been used consistently to measure resilience among diverse groups of the population. The value for Cronbach’s alpha in this study was excellent at α = 0.92. While two further versions of the CD-RISC have been developed involving two items [40] and ten items [41], this survey used the original CD-RISC [38] which referred to the 25-item survey. The items are scored on a 5-point Likert scale from ‘not true at all’ to ‘true nearly all of the time’. The scale relates to how the respondents have felt in the last month and the total scores range from 1 to 100.

#### Work-SoC [34, 42]

This is a nine-item scale consisting of three subscales. Participants rate on a seven-point semantic differential scale each with bipolar adjective pairs (e.g. controllable versus uncontrollable) a series of statements covering the three dimensions of meaningfulness, comprehensibility, and manageability. Scores were calculated for each of the dimensions and those with higher scores indicated a higher work sense of coherence. Mean scores of each item were calculated as total scores. Cronbach’s alpha for the present study was α = 0.93 demonstrating excellent internal consistency.

#### Demographics and work characteristics

While there is debate in the literature as to the impact of demographic variables on resilience [37–39]. Data on potential predictors such as age, gender, ethnicity, education, and years of caring experience were collated along with work characteristics (statutory/independent, place of work, employment type and role).

### Data analysis

Survey results were downloaded for analysis into IBM-SPSS (Statistical Package for the Social Sciences) version 28. Normality testing of data determined which test (parametric or non-parametric) to use. A series of descriptive and inferential tests were undertaken to describe participant characteristics and determine if a relationship existed between independent variables and resilience. In this study the statistical significance was set at 0.05. We report descriptive statistics of the participating sample, mean resilience scores, how they measured across demographic variables and explored if a relationship existed between work sense of coherence scores and resilience scores. Bivariate analysis was undertaken on variables that have been deemed significant in other relevant studies.

## Results

In total 613 valid data sets formed the basis of the analysis with 585 participants completing the first two sections: the CDRISC and the Work-SoC, with 573 participants completing all three sections of the survey. As of February 2023, there were an estimated 37,000 social care workers working in Northern Ireland, this result represents 1.7% of that population. While all registrants were sent the email, we have no record of those who opened the email to respond.

### Demographic and work characteristics of the survey respondents

Descriptive analysis revealed a diverse sector in terms of job title and context (See Table 1). As well as care assistants, domiciliary care workers and managers, participants self-identified as support workers across different disciplines of Health, Allied Health, and Social Work. Titles were reported according to context such as day care workers, community care workers, some identified as project workers and home care workers. 43% of the respondents were working for one of the Health and Social Care Trusts with almost 49% working for the independent sector. 8% were deemed as ‘other’, and this included charities, other voluntary organisations such as homeless shelters. While 75% of the respondents identified as working in a care home/residential facility or in domiciliary care, the place of work also included day care, community support, charities, homeless services, and supported living.

The mean age of participants was 45 (SD 11.8),which is slightly higher than the mean age of 42 recorded by NISCC. 20% of respondents were male. The Northern Ireland Social Care Council (NISCC) record an overall 16% male registrants, representing 9% in domiciliary care, 19% in adult residential care and 26% in supported living, given that 37% in the current study were from domiciliary care, this might explain the increase in male participation. While we analysed the age variable as a continuous variable, we have presented it in categories to reflect representation in line with the distribution across the sample 89% of respondents were White. Other ethnicities included: Black African (4%), Indian (2%), mixed ethnic (2%) another ethnicity (2%) and Filipino (1%). While the Northern Ireland Social Care Council requests information on ethnicity it is not mandatory, of the 60% that completed this section at registration, 84% identified as Caucasian, which is lower than the 89% reported in this sample. This difference may be attributed to factors such as language barriers or the accessibility and ease of completing online surveys. The distribution of ‘other ethnicities’ in the survey closely mirrored those recorded by NISCC with Black African, Indian, Filipino and mixed ethnic groups representing the largest proportions after Caucasian. The difference in mean resilience scores between white and other ethnicities did not reach statistical significance. While only limited information relating to the education levels of social care workers is presently available this cohort reported higher levels of education. Nine percent of the respondents were in the job less than a year with 16% being in the job for 23 years or longer. 33% of respondents in the study had attained a Batchelor’s degree or higher level of education. 63% were married, and the majority worked full time (71%).

### Resilience Scores including demographic and work characteristics

A mean resilience score of 70.4 (SD 13.3) on the CD-RISC was reported on 613 responses.

Table 1 presents the resilience scores across the demographic variables and work characteristics of the participating sample. There were 573 respondents who completed the demographic and work characteristic section upon which this analysis is based.

**Table 1:** Demographic and work characteristics with mean resilience scores on CD-RISC

Variable	Frequency (%)	Mean CD-RISC (SD)	*P* value
***Gender***			
Female	458 (80%)	71.5 (12.8)	
Male	115 (20%)	66.4 (16.1)	0.002
***Ethnicity***			
* White*	510 (89%)	70.2 (13.6)	
* Other ethnicity* ^*^	63 (11%)	72.8 (13.8)	*0.161*
***Age categories***			
17yrs – 30years	79 (14%)	67.1 (13.1)	
31 yrs. − 50 years.	288 (50%)	70.5 (13.5)	
51 yrs. – 70years	206 (36%)	71.8 (14.0)	0.038
***Education Level***			
* Below Batchelor level*	384 (67%)	69.6 (13.3)	0.029
* Batchelor Degree +*	189 (33%)	72.3 (14.1)	
Years of Experience			
< 10years	319 (56%)	70.6 (13.0)	
> 10years	254 (44%)	70.3 (14.5)	0.771
***Year of experience – categories***			
< 1year	49 (9%)	75.4 (11.9)	
1-5years	152 (27%)	71.3 (12.7)	
6-10years	118 (21%)	67.8 (13.3)	0.015 with < 1yr
11-16years	86 (15%)	72.2 (14.4)	
17-22years	75 (13%)	69.0 (15.8)	
> 23years	93 (16%)	69.6 (13.4)	
***Marital status***			
Single/divorced/widowed/separated	214 (37%)	69.2 (12.7)	0.075
Married/Domestic partnership	359 (63%)	71.3 (14.2)	
***Employment***			
Full-Time	405 (71%)	70.6 (14.1)	
Part Time	168 (29%)	70.3 (12.6)	0.836
***Workplace***			
NI (Northern Ireland) Health and Social Care Trusts	246 (43%)	70.2 (13.6)	0.831
Independent Health Care	280 (49%)	70.8 (13.7)	
Other	47 (8%)	70.0 (14.0)	
***Place of Care***			
Care Home/Residential Facility	216 (38%)	69.5 (14.9)	
Domiciliary Care	212 (37%)	71.7 (12.5)	
Other	145 (24%)	70.2 (13.7)	0.230
***Care Role***			
Care Assistant	261 (45%)	70.2 (13.5)	
Senior care assistant	126 (22%)	71.2 (13.9)	
Managerial Role	80 (14%)	69.8 (15.6)	
Other	106 (19%)	71.0 (13.7)	0.846

*Other ethnicities included, Black African, Indian, Mixed Ethnic, Irish, Irish Traveller, Roma, Other Black ethnic group, Nepalese, Sri Lankan, Kazakhstan, White African

Significance level *p* ≤ 0.05 Percentages are rounded to whole numbers.

A significant effect of gender on resilience was noted with males reporting lower levels of resilience (*p* = 0.002). The mean age of this sample was 45 (SD = 11.8). Our findings showed that as participants got older their resilience tended to increase (*p* = 0.046). We found that individuals with higher levels of education had statistically significant higher resilience scores (*p* = 0.029). When examining resilience scores as a dichotomous variable, no significant differences were found between individuals with less than 10 years’ experience and those with over 10 years’ experience (*p* = 0.77). However, within categories a distinction emerged between those 6-10yrs experience and those with less than one year. Individuals with less than one years’ experience (*n* = 49) exhibited significantly higher resilience scores (*p* = 0.015). There were no significant differences in resilience levels reported between those working for statutory and non-statutory organisations. No differences in mean resilience scores were noted as to the place of care. There was a lower level of resilience recorded for those in a managerial role however this did not reach statistical significance.

### Work-related Sense of Coherence including demographic and work characteristics

The mean Work-SoC (*n* = 585) was calculated 4.7 (SD 1.5), significant differences were noted in gender with males reporting a lower Work-related Sense of Coherence (*p* = 0.005). Those who worked part time reported higher levels of Work- SoC (*p* = 0.019). See Table 2 below. There were no other significant differences reported.

**Table 2:** Significant demographic variables related to Work-SoC

Variable	Frequency (%)	Mean WORK- SoC (SD)	*P* value
***Gender***			
Male	115 (20%)	4.3 (1.6)	0.005
Female	458 (80%)	4.8 (1.5)	
***Employment***			
Full-Time	405 (71%)	4.6 (1.5)	
Part Time	168 (29%)	4.9 (1.5)	0.019

The relationship between Work-related Sense of Coherence and Resilience.

There was a moderate positive relationship between resilience and Work-SoC (*r* = 0.400, *p* < 0.001, *n* = 585) suggesting that more resilient individuals may be more likely to find their work more meaningful, manageable, and understandable and that those who find their work more meaningful, manageable, and understandable would be better able to positively manage future challenges.

## Discussion

This study is novel in exploring the concept of resilience as it relates to social care workers in Northern Ireland. While we have no population statistics for social care workers and the CD-RISC is known to be influenced by both region and nature of population [Davidson JRT. Connor-Davidson Resilience Scale (CDRISC) Manual unpublished. 06-23-2021], we can look to studies with other groups for comparison. Resilience scores in this study (M = 70.4 SD 13.3) may be considered in the lowest quartile if viewed according to a United States general population study undertaken by the CD-RISC scale authors (M = 80.4, SD = 12.8) [38]. A UK population-based study using a representative sample undertaken in an NHS trust (*n* = 1006) used the Connor-Davidson Resilience 10 item scale [41] to measure resilience in the psychometric testing of an English Coping Scale [43]. While a different population the resilience level was also reported to be in the lowest quartile as outlined in the original scale validation [41]. Comparing mean resilience scores recorded on general populations in Hong Kong [44] (M = 60, SD = 13.9), Italy [45] (M = 66.7 SD = 12.4) and Sweden [46] (M = 68.8, SD = 13), the resilience level in this study is higher.

A meta-analysis of resilience among the general population and health care professionals measured during the COVID-19 pandemic demonstrated a global low resilience in both these populations (data 2020–2022) [47]. Given the data for this study was collected shortly after restrictions imposed during the pandemic were lifted, the findings may suggest that despite the intense challenges endured in the pandemic social care workers maintained a moderate resilience level. In a pilot study undertaken by the study authors [16] with a small sample of care home nurses and managers (*n* = 56) the resilience level was reported higher at 77.3. The differences between populations could be explained by many factors; social care workers may perceive their role as having less value and status than nurses and managers [48]. This coupled with fragmented systems, lack of resources, zero contract hours and weak career paths [49] may contribute to low resilience and feelings of devaluation. Indeed, it has been suggested that the scheduling and allocation of resources in a symbolic way can demarcate who and what is valued in society [44].

However, the mean resilience score in this study was higher than the findings from a study undertaken measuring resilience of medical doctors across the UK [50]. Doctors from Northern Ireland had a mean resilience score of 68.5 (SD = 12) while those from England reported lower scores (M = 64, SD = 12) with Northern Ireland reporting the highest score of the four Nations. The sense of connection and confidence afforded by the registering body for social care in Northern Ireland (NISCC) may have impacted resilience levels in this sample of social care workers as Northern Ireland was the first of the four regions to have complete registration of the domiciliary care workforce and an evaluation of the process [1]. Social care workers have experienced many pre-existing systemic challenges within the social care system [6] where adaptation, resourcefulness and balancing of job resources have been a necessary part of everyday work. In the privileged position of caring for people in their community environments social care workers may see their positive relationship with clients as building resilience. Mutually rewarding relationships where the client receives quality care and the care worker felt they were making a difference was a key finding in a study exploring resilience in domiciliary care workers in the UK [17].

### Factors shaping resilience among social care workers

A significant finding that male social care workers reported lower resilience levels and lower levels of work-related sense of coherence warrants further enquiry. Despite the different context this finding is in keeping with a study measuring resilience in staff employed in one NHS Trust in the United Kingdom (*n* = 845) that included management, clinical ancillary, and administrative staff [51]. However, the finding of prevalence of lower resilience in males is contrary to the findings of a meta-analysis of studies on gender and resilience undertaken in 2021 [52] that reported the prevalence of low resilience was higher in females. A recent study investigating well-being in health and social care in the UK during the COVID pandemic reported males as having higher well-being than females [6]. Many of the studies relating to resilience in health care workers are undertaken with nurses, doctors and allied health professionals, gendered differences are highlighted relating to these groups. Yet there is a dearth of literature relating to gender and social care workers; indeed, we know little as to why men are under-represented in care work.

There are many potential reasons for the finding that males in this study reported lower levels of resilience. The importance of social connection and support proliferates the resilience literature [53, 54], with findings that patterns of social connectedness among men are diverse [55]. Female workers tend to report finding resilience factors in social support more frequently than male colleagues [54]. There has been some debate in the literature relating to the need for a more gendered look at resilience and its measurement and the lack of consideration of roles, expectations and environmental factors which shape experiences and response to adversity [56].

A qualitative study exploring the experiences and perceptions of male workers in social care in Spain (*n* = 31) found that males working in social care have to negotiate societal expectations and norms of masculinities balanced with having to get a job and being in one that is considered female and low paid [57]. In a study examining career patterns of low- and middle-skill men in health care occupations, it was found that males who were involved in direct care of people earned less compared to men in other occupations after controlling for demographic characteristics [58]. The authors concluded that the ‘glass ceiling’ effect of rising wages due to males undertaking previously considered female work did not apply in jobs that involved direct caring.

In this study, findings revealed that older individuals and those with higher levels of education reported higher levels of resilience as measured by the CD-RISC. This mirrors the findings of studies with diverse groups of people working in health care roles [59–62]. People who are older may have faced more life challenges and may have had opportunities to develop problem-solving capabilities having learnt how to negotiate for resources, deal with uncertainty and safely navigate risky and uncertain situations that lie beyond their control. It was interesting to note that those individuals who were in the job less than a year (*n* = 49) had significantly higher levels of resilience. An explanation is that new employees may have a stronger sense of purpose and motivation, as they are still in the early stages of their career and eager to prove themselves. Additionally, new employees may have received more recent training and education, which could contribute to their resilience and motivation in joining the social care workforce.

The finding that those with higher levels of education reported higher levels of resilience mirrors other study findings [60, 63]. A radical reform of education conferring professional status on social care workers has been advocated [64]. However, in isolation to organisational policy and societal change this may be of little benefit. Having a professional status without the commiserate pay and conditions could drive workers further away from social care. The influence of demographics on resilience has been found to be inconsistent when measured in other populations [65]. However a supportive work environment, one that encourages a healthy work-life balance, minimises isolation and promotes cohesion and collegiality has been consistently identified as a key element in building both resilience and wellbeing [53, 66].

### Work sense of coherence and resilience

In this study the mean Work-SoC of 4.7 (SD = 1.6) was slightly lower than the mean score of 4.9 (SD = 1) recorded by the scale authors [34, 42]. The scale is considered to be consistent and not influenced by demographic differences [34]. However, two notable differences were found in our sample in that males scored lower than females and those who worked full time scored lower than part time. The reasons for these differences may relate to the gender issues already identified and the perception of inequity and lower value related to social care work. The finding that part time work was associated with higher Work-SOC but not resilience warrants further enquiry as the concept has been shown to be a valuable indicator of health-related quality of working life [42]. This finding may be due to part time workers feeling they can maintain a better work life balance with more control over their hours of work allowing them to better deal with work stressors. The findings from the current study suggest that work perceived as manageable, and meaningful may increase resilience as evidenced by a moderate positive association between Work-SoC and resilience. In a longitudinal study of nursing home employees higher Work-SoC was related to commitment and dedication to future work [35]. A nurturing workplace environment has been shown to reduce the emotional and psychological impact of stressors whereas the perception of an environment that is stressful and unmanageable can increase distress [67]. A satisfactory working environment was found to improve new nurses’ resilience reducing their intent to leave their workplace. This suggests that new employees who feel supported and engaged in their work may have higher levels of resilience [68].

**Fig. 1:**
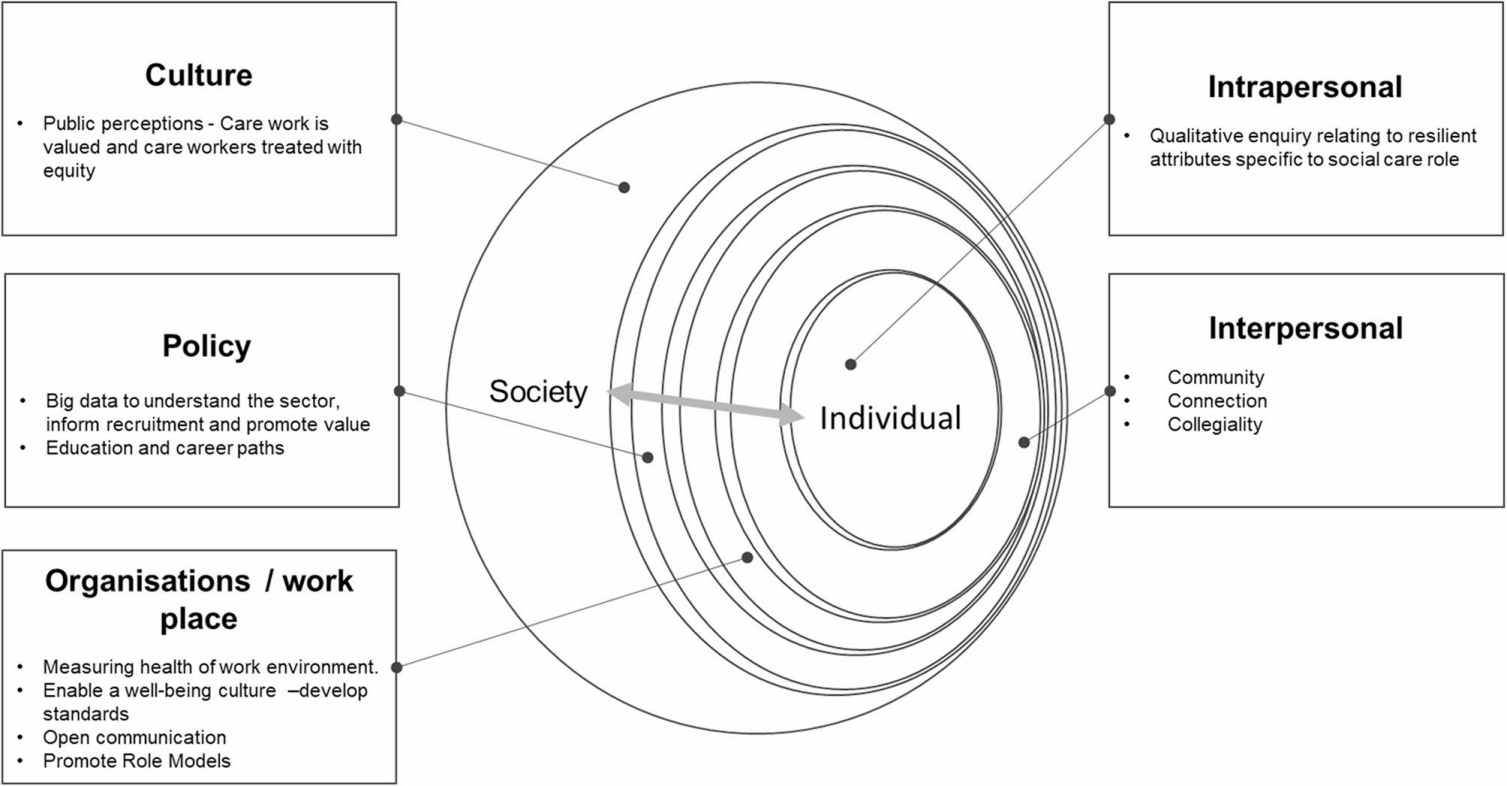
Future Directions for practice and research using a socio-ecological systems approach to resilience building in social care

Figure 1 below depicts the concentric and embedded levels of the socio ecological model as it applies to this study [33, 69] with potential implications for practice and policy. A starting point at the individual level is to gather comprehensive demographic and operational data to understand the social care workforce and support them across their diverse roles. At an organisational level the potential of novice social care workers should be developed capitalising on the assets of those more expert members of the workforce. Supporting the development and nurturing of resilience among social care workers may have the potential to influence work engagement providing more value, understanding and perceived capability in doing the job. The bidirectional outcomes of developing agency at the individual level may empower social care workers to proactively seek resources, to advocate for better working conditions, thereby influencing a societal shift in attitude to one that considers social care work a valued career for people of all genders.

### Strengths and limitations

Strengths of this research include its comprehensive sampling strategy, which aimed to encompass the diverse and geographically dispersed population of social care workers in Northern Ireland. The study employed a census sampling strategy, reaching out to all registered social care workers through the Northern Ireland Social Care Council (NISCC), thereby minimising sampling bias. Additionally, the use of validated measurement tools such as the Connor-Davidson Resilience Scale (CD-RISC) and the Work-related Sense of Coherence Scale (Work-SoC) enhances the validity of the findings. The study’s quantitative approach allows for the analysis of relationships between variables and provides statistical significance to the results, offering valuable insights into the factors influencing resilience among social care workers.

However, there are notable limitations to consider. Firstly, the small proportion of the sample, representing only 1.7% of the estimated 37,000 social care workers in Northern Ireland, raises questions about the generalisability of the findings to the broader population. Further work should look to uncover ways to engage people working in social care to participate in research. Additionally, the cross-sectional design of the study, capturing data at a single point in time, limits the ability to establish causal relationships or capture changes in resilience over time. Furthermore, while efforts were made to ensure diversity in the sample, certain subgroups of social care workers may be underrepresented, potentially biasing the results. The possible over representation of participants with higher levels of education could mean that the perspectives of those with lower levels of education may be underrepresented. Future research might consider a stratified sampling technique to ensure representation across education levels. Finally, the reliance on self-report measures introduces the possibility of response bias and social desirability effects, impacting the accuracy of the data collected. However, this study is novel in exploring the relationship between the work environment and resilience in this population of social care workers.

## Conclusion

This study aimed to explore and better understand resilience and associated factors within the social care workforce in Northern Ireland. The findings of this study suggest scope for a multi-level and all systems approach to both sustaining and nurturing resilience. An approach that recognises the diverse and asset rich makeup of the work force and the responsibility of organisations to develop supportive work environments. This approach should focus on building capabilities to find meaning, navigate challenges, and thrive in the work environment.

Further research should aim to explore the motivation to choose social care work and what factors enable and hinder people when considering a job in social care. Given the mean age of this workforce, it would be valuable to consider the perceptions of social care held by young adults who are making career choices. Focusing on the assets within the system and capitalising on these to attract new workers should be part of a public health drive to positively influence public perception of social care and challenge societal stereotypes that depict care as primarily a female domain and one that is afforded less value.

## Supplementary Information

Supplementary Material 1.

## Data Availability

The datasets used and analysed during the current study are available from the corresponding author on reasonable request.

## Abbreviations

CD-RISC: Connor-Davidson Resilience Scale
Work-SoC: Work related Sense of Coherence Scale
SD: Standard Deviation
NI: Northern Ireland
UK: United Kingdom
STROBE: STrengthening the Reporting of OBservational studies in Epidemiology
NISSC: Northern Ireland Social Care Council
IBM-SPSS: IBM Statistical Package for the Social Sciences

## References

[1] Dodsworth E, Oung C, Nuffield Trust. ‘What does the social care workforce look like across the four countries?‘, in Adult social care in the four countries of the UK. Explainer series,. 2023 [Available from: https://www.nuffieldtrust.org.uk/news-item/what-does-the-social-care-workforce-look-like-across-the-four-countries0

[2] Johnson M, Rubery J, Egan M. Raising the bar? The impact of the UNISON ethical care campaign in UK domiciliary care. Transfer: Eur Rev Labour Res. 2021;27(3):367–82.

[3] Marshall F, Gordon A, Gladman JRF, Bishop S. Care homes, their communities, and resilience in the face of the COVID-19 pandemic: interim findings from a qualitative study. BMC Geriatr. 2021. 10.1186/s12877-021-02053-9. 33546612 10.1186/s12877-021-02053-9 PMC7863040

[4] Nyashanu M, Pfende F, Ekpenyong MS. Triggers of mental health problems among frontline healthcare workers during the COVID-19 pandemic in private care homes and domiciliary care agencies: Lived experiences of care workers in the Midlands region. UK Health Soc Care Community. 2022;30(2):e370–76. 10.1111/hsc.13204. 33107131 10.1111/hsc.13204

[5] Jordan J-A, Shannon C, Browne D, Carroll E, Maguire J, Kerrigan K, et al. COVID-19 staff wellbeing survey: longitudinal survey of psychological well-being among health and social care staff in Northern Ireland during the COVID-19 pandemic. BJPsych Open. 2021. 10.1192/bjo.2021.988. 34493960 10.1192/bjo.2021.988 PMC8410744

[6] Mallett J, Nicholl P, Neill R, Manthorpe J, Moriarty J, Schroder H, Curry D. Working conditions and well-being in UK social care and social work during COVID-19. J Soc Work (Lond). 2023;23(2):165–88. 10.1177/14680173221109483. 38603207 10.1177/14680173221109483 PMC9264376

[7] Mallon A, Mitchell G, Carter G, McLaughlin D, Wilson CB. A rapid review of evaluated interventions to inform the development of a resource to support the resilience of care home nurses. BMC Geriatr. 2023;23(1):275. 37147594 10.1186/s12877-023-03860-y PMC10162002

[8] Kunzler AM, Helmreich I, Chmitorz A, Konig J, Binder H, Wessa M, et al. Psychological interventions to foster resilience in healthcare professionals. Cochrane Database Syst Reviews. 2020;7:CD012527. 10.1002/14651858.CD012527.pub2 PMC8121081 32627860

[9] Pearson C, Watson N, Brunner R, Cullingworth J, Hameed S, Scherer N, et al. Covid-19 and the crisis in social care: exploring the experiences of disabled people in the pandemic. Social Policy Soc. 2023;22(3):515–30.

[10] Elliott KEJ, Stirling CM, Martin AJ, Robinson AL, Scott JL. We are not all coping: a cross-sectional investigation of resilience in the dementia care workforce. Health Expect. 2016;19(6):1251–64. 26472594 10.1111/hex.12419 PMC5139051

[11] Fletcher D, Sarkar M. Psychological resilience: a review and critique of definitions, concepts, and theory. Eur Psychol. 2013;18(1):12.

[12] Rutter M. Resilience: Causal pathways and social ecology. In M. Ungar (Ed.), The social ecology of resilience: A handbook of theory and practice. Springer Science + Business Media; 2012. p. 33–42. 10.1007/978-1-4614-0586-3_3.

[13] Deldar K, Froutan R, Dalvand S, Gheshlagh RG, Mazloum SR. The relationship between resiliency and burnout in Iranian nurses: A systematic review and Meta-Analysis. Open Access Macedonian J Med Sci. 2018;6(11):2250–6. 10.3889/oamjms.2018.428 PMC6290412 30559897

[14] Ang SY, Hemsworth D, Uthaman T, Ayre TC, Mordiffi SZ, Ang E, et al. Understanding the influence of resilience on psychological outcomes — comparing results from acute care nurses in Canada and Singapore. Appl Nurs Res. 2018;43:105–13. 30220356 10.1016/j.apnr.2018.07.007

[15] Finstad GL, Giorgi G, Lulli LG, Pandolfi C, Foti G, León-Perez JM, et al. Resilience, coping strategies and posttraumatic growth in the workplace following COVID-19: a narrative review on the positive aspects of trauma. Int J Environ Res Public Health. 2021;18(18): 9453. 34574378 10.3390/ijerph18189453 PMC8468098

[16] Mallon A, Mitchell G, Carter G, Francis McLaughlin D, Linden M, Brown Wilson C. Exploring resilience in care home nurses: an online survey. Healthcare. 2023;11(24):3120. 38132010 10.3390/healthcare11243120 PMC10742816

[17] Donnellan WJ, Hirons A, Clarke K, Muinos C, McCabe L. Exploring resilience in UK-based domiciliary care workers before and during the COVID-19 pandemic. Int J Environ Res Public Health. 2022;19(23): 16128. 36498201 10.3390/ijerph192316128 PMC9739467

[18] Guo Y-F, Cross W, Plummer V, Lam L, Luo Y-H, Zhang J-P. Exploring resilience in Chinese nurses: a cross-sectional study. J Nurs Adm Manag. 2017;25(3):223–30. 10.1111/jonm.12457 28164403

[19] Han JE, Park NH, Cho J. Influence of gender role conflict, resilience, and nursing organizational culture on nursing work performance among clinical nurses. J Korean Acad Soc Nurs Educ. 2020;26(3):248–58.

[20] Mealer M, Jones J, Newman J, McFann KK, Rothbaum B, Moss M. The presence of resilience is associated with a healthier psychological profile in intensive care unit (ICU) nurses: results of a national survey. Int J Nurs Stud. 2012;49(3):292–9. 21974793 10.1016/j.ijnurstu.2011.09.015 PMC3276701

[21] Leng M, Xiu H, Yu P, Feng J, Wei Y, Cui Y, et al. Current state and influencing factors of nurse resilience and perceived job-related stressors. J Continuing Educ Nurs. 2020;51(3):132–7. 10.3928/00220124-20200216-08 32119108

[22] Mc Gee SL, Höltge J, Maercker A, Thoma MV. Sense of coherence and stress-related resilience: investigating the mediating and moderating mechanisms in the development of resilience following stress or adversity. Front Psychiatry. 2018;9:378. 30186189 10.3389/fpsyt.2018.00378 PMC6110848

[23] Antonovsky A. Unraveling the mystery of health: how people manage stress and stay well. San Francisco, CA, USA: Jossey-bass; 1987.

[24] Eriksson M, Mittelmark MB. The sense of coherence and its measurement. Springer International Publishing. In: Mittelmark MB, Sagy S, Eriksson M, Bauer GF, Pelikan JM, Lindström B, Espnes GA, editors. The Handbook of Salutogenesis [Internet]. Cham: Springer; 2017. p. 97–106. 10.1007/978-3-319-04600-6_12.

[25] Vaandrager L, Kennedy L. The application of salutogenesis in communities and neighborhoods. In Mittelmark MB, Bauer GF, Vaandrager L, et al, editors. The handbook of salutogenesis. Cham: Springer; 2017:159 – 70. Available: https://www.ncbi.nlm.nih.gov/books/NBK584108/.

[26] Mittelmark MB. Resilience in the salutogenic model of health. Ed: Ungar, M. Multisystemic Resilience: Adaptation and Transformation in Contexts of Change. United States: Oxford University Press; 2021. p. 153–64. https://www.google.co.uk/books/edition/_/5LscEAAAQBAJ?hl=en&gbpv=1.

[27] Eriksson M, Contu P. The Sense of Coherence: Measurement Issues. In: Mittelmark MB, Bauer GF, Vaandrager L, Pelikan JM, Sagy S, Eriksson M, Lindström B, Meier Magistretti C, editors. The Handbook of Salutogenesis [Internet]. 2nd ed. Cham: Springer; 2022. p. 79–91. 10.1007/978-3-030-79515-3_11. 36121979

[28] Navarro Prados AB, Jiménez García-Tizón S, Meléndez JC. Sense of coherence and burnout in nursing home workers during the COVID‐19 pandemic in Spain. Health Soc Care Commun. 2022;30(1):244–52. 10.1111/hsc.13397 PMC8250978 33894094

[29] Ungar M. The social ecology of resilience: a handbook of theory and practice. New York: Springer Science & Business Media; 2011.

[30] Grimes C. Leading Change in Health and Social Care: Building Relationships, Diversity and Action. By Corrina Grimes Jan 22nd 2024 [Available from: https://blogs.bmj.com/bmjleader/2024/01/22/leading-change-in-health-and-social-care-building-relationships-diversity-and-action-by-corrina-grimes/

[31] Fleming G, Taylor BJ. Battle on the home care front: perceptions of home care workers of factors influencing staff retention in Northern Ireland. Health Soc Care Community. 2006;15(0):67–76. 10.1111/j.1365-2524.2006.00666.x 17212627

[32] Merrill RM, Aldana SG, Pope JE, Anderson DR, Coberley CR, Grossmeier JJ, et al. Self-rated job performance and absenteeism according to employee engagement, health behaviors, and physical health. J Occup Environ Med. 2013;55(1):10–8. 23254387 10.1097/JOM.0b013e31827b73af

[33] Simons-Morton B, McLeroy K, Wendel M. Behavior theory in health promotion practice and research. Burlington, MA, USA: Jones & Bartlett; 2012.

[34] Vogt K, Jenny GJ, Bauer GF. Comprehensibility, manageability and meaningfulness at work: construct validity of a scale measuring work-related sense of coherence. SA J Ind Psychol. 2013;39(1):1–8.

[35] Grødal K, Innstrand ST, Haugan G, André B. Work-related sense of coherence and longitudinal relationships with work engagement and job satisfaction. Scand J Work Organ Psychol. 2019;4(1):Article 5. 10.16993/sjwop.73.

[36] Van der Westhuizen SC. Incremental validity of work-related sense of coherence in predicting work wellness. SA Journal of Industrial Psychology/SA Tydskrif vir Bedryfsielkunde. 2018;44(0):a1467. 10.4102/sajip.v44i0.1467.

[37] Berger-Estilita J, Abegglen S, Hornburg N, Greif R, Fuchs A. Health-promoting quality of life at work during the COVID-19 pandemic: a 12-month longitudinal study on the work-related sense of coherence in acute care healthcare professionals. Int J Environ Res Public Health. 2022;19(10): 6053. 35627590 10.3390/ijerph19106053 PMC9140864

[38] Connor KM, Davidson JRT. Development of a new resilience scale: the Connor-Davidson resilience scale (CD-RISC). Depress Anxiety. 2003;18(2):76–82. 12964174 10.1002/da.10113

[39] Von Elm E, Altman DG, Egger M, Pocock SJ, Gøtzsche PC, Vandenbroucke JP. The strengthening the reporting of observational studies in epidemiology (STROBE) statement: guidelines for reporting observational studies. Lancet. 2007;370(9596):1453–7. 18064739 10.1016/S0140-6736(07)61602-X

[40] Vaishnavi S, Connor K, Davidson JRT. An abbreviated version of the Connor-Davidson resilience scale (CD-RISC), the CD-RISC2: psychometric properties and applications in psychopharmacological trials. Psychiatry Res. 2007;152(2–3):293–7. 17459488 10.1016/j.psychres.2007.01.006 PMC2041449

[41] Campbell-Sills L, Stein MB. Psychometric analysis and refinement of the connor–davidson resilience scale (CD-RISC): validation of a 10-item measure of resilience. J Trauma Stress. 2007;20(6):1019–28. 18157881 10.1002/jts.20271

[42] Bauer GF, Vogt K, Inauen A, Jenny GJ. Work-SoC–Entwicklung und validierung einer Skala Zur erfassung des arbeitsbezogenen Kohärenzgefühls. Z Für Gesundheitspsychologie. 2015;23(1):20–30.

[43] O’Rourke T, Budimir S, Pieh C, Probst T. Psychometric qualities of the English coping scales of the stress and coping inventory in a representative UK sample. BMC Psychol. 2021;9:1–11. 33531087 10.1186/s40359-021-00528-3 PMC7851809

[44] Ni MY, Li TK, Yu NX, Pang H, Chan BH, Leung GM, et al. Normative data and psychometric properties of the Connor–Davidson resilience scale (CD-RISC) and the abbreviated version (CD-RISC2) among the general population in Hong Kong. Qual Life Res. 2016;25:111–6. 26198665 10.1007/s11136-015-1072-x

[45] Bonaccio M, Di Castelnuovo A, Costanzo S, Pounis G, Persichillo M, Cerletti C, et al. Mediterranean-type diet is associated with higher psychological resilience in a general adult population: findings from the Moli-sani study. Eur J Clin Nutr. 2018;72(1):154–60. 28952609 10.1038/ejcn.2017.150

[46] Velickovic K, Rahm Hallberg I, Axelsson U, Borrebaeck CA, Rydén L, Johnsson P, et al. Psychometric properties of the Connor-Davidson resilience scale (CD-RISC) in a non-clinical population in Sweden. Health Qual Life Outcomes. 2020;18:1–10. 32398074 10.1186/s12955-020-01383-3 PMC7216522

[47] Janitra FE, Jen H-J, Chu H, Chen R, Pien L-C, Liu D, et al. Global prevalence of low resilience among the general population and health professionals during the COVID-19 pandemic: a meta-analysis. J Affect Disord. 2023;332:29–46. 37004902 10.1016/j.jad.2023.03.077 PMC10063525

[48] Saloniki E-C, Turnpenny A, Collins G, Marchand C, Towers A-M, Hussein S. Abuse and wellbeing of long-term care workers in the COVID-19 era: evidence from the UK. Sustainability. 2022;14(15):9620.

[49] Curry N, Oung C, Hemmings N, Comas-Herrera A, Byrd W. Building a resilient social care system in England. London, UK: Nuffield Trust; 2023. pp. 2023–05.

[50] McKinley N, McCain RS, Convie L, Clarke M, Dempster M, Campbell WJ, et al. Resilience, burnout and coping mechanisms in UK doctors: a cross-sectional study. BMJ Open. 2020;10(1):e031765. 31988223 10.1136/bmjopen-2019-031765 PMC7045750

[51] Sull A, Harland N, Moore A. Resilience of health-care workers in the UK; a cross-sectional survey. J Occup Med Toxicol. 2015;10:1–8. 26029246 10.1186/s12995-015-0061-x PMC4449529

[52] Ayşe G, KOĞAR EY. A meta-analysis study on gender differences in psychological resilience levels. Kıbrıs Türk Psikiyatri Ve Psikoloji Dergisi. 2021;3(2):132–43.

[53] Huey CWT, Palaganas JC. What are the factors affecting resilience in health professionals? A synthesis of systematic reviews. Med Teach. 2020;42(5):550–60. 31984844 10.1080/0142159X.2020.1714020

[54] Gillman JC, Turner MJ, Slater MJ. The role of social support and social identification on challenge and threat cognitive appraisals, perceived stress, and life satisfaction in workplace employees. PLoS One. 2023;18(7):e0288563. 37437025 10.1371/journal.pone.0288563 PMC10337949

[55] McKenzie SK, Collings S, Jenkin G, River J. Masculinity, social connectedness, and mental health: men’s diverse patterns of practice. Am J Men’s Health. 2018;12(5):1247–61. 29708008 10.1177/1557988318772732 PMC6142169

[56] Hirani S, Lasiuk G, Hegadoren K. The intersection of gender and resilience. J Psychiatr Ment Health Nurs. 2016. 10.1111/jpm.12313. 27593204 10.1111/jpm.12313

[57] Puerta YB, d’Argemir DC, Escoda MR. What i really want is a job’. Male workers in the social care sector. Masculinidades Y Cambio Social. 2020;9(2):207–34.

[58] Dill JS, Price-Glynn K, Rakovski C. Does the glass escalator compensate for the devaluation of care work occupations? The careers of men in low-and middle-skill health care jobs. Gend Soc. 2016;30(2):334–60.

[59] Gillespie BM, Chaboyer W, Wallis M. The influence of personal characteristics on the resilience of operating room nurses: a predictor study. Int J Nurs Stud. 2009;46(7):968–76. 17915223 10.1016/j.ijnurstu.2007.08.006

[60] Ang SY, Uthaman T, Ayre TC, Mordiffi SZ, Ang E, Lopez V. Association between demographics and resilience - a cross-sectional study among nurses in Singapore. Int Nurs Rev. 2018;65(3):459–66. 29517143 10.1111/inr.12441

[61] Zheng Z, Gangaram P, Xie H, Chua S, Ong SBC, Koh SE. Job satisfaction and resilience in psychiatric nurses: a study at the Institute of Mental Health, Singapore. Int J Ment Health Nurs. 2017;26(6):612–9. 28160378 10.1111/inm.12286

[62] Mealer M. Factors affecting resilience and development of post traumatic stress disorder in critical care nurses. Am J Crit Care. 2017;26(3):184–92. 28461539 10.4037/ajcc2017798 PMC5685839

[63] Guo YF, Cross W, Plummer V, Lam L, Luo YH, Zhang JP, John Wiley Sons Inc. Exploring resilience in Chinese nurses: a cross-sectional study. J Nurs Manage. 2017;25(3):223–30. 10.1111/jonm.12457 28164403

[64] Wild DJ, Szczepura A. Reimagining care homes: can the COVID-19 pandemic act as a catalyst for enhancing staff status and education? Nurs Older People. 2021;33(5):20–5. 10.7748/nop.2021.e1321. 10.7748/nop.2021.e1321 34008354

[65] Yu F, Raphael D, Mackay L, Smith M, King A. Personal and work-related factors associated with nurse resilience: a systematic review. Int J Nurs Stud. 2019;93:129–40. 30925279 10.1016/j.ijnurstu.2019.02.014

[66] Silver JK, Ellinas EH, Augustus-Wallace AC. Sense of belonging is a critical component of workforce retention. BMJ. 2024;384:q392. 38365283 10.1136/bmj.q392

[67] Hines SE, Chin KH, Glick DR, Wickwire EM. Trends in moral injury, distress, and resilience factors among healthcare workers at the beginning of the COVID-19 pandemic. Int J Environ Res Public Health. 2021;18(2): 488. 33435300 10.3390/ijerph18020488 PMC7826570

[68] Park K, Jang A. Factors affecting the resilience of new nurses in their working environment. Int J Environ Res Public Health. 2022;19(9): 5158. 35564552 10.3390/ijerph19095158 PMC9102416

[69] Mallon A, Hasson F, Casson K, McIlfatrick S. Young adults understanding and readiness to engage with palliative care : extending the reach of palliative care through a public health approach: a qualitative study. BMC Palliative Care. 2021;20(1):120. 10.1186/s12904-021-00808-0 PMC8320215 34320961

